# Frontal Variant Alzheimer’s Disease or Primary Psychiatric Disorder? A Case Report

**DOI:** 10.3390/reports8010024

**Published:** 2025-02-18

**Authors:** Siew Fai Liew, Weishan Li

**Affiliations:** 1 Department of Psychiatry, Sengkang General Hospital, 110 Sengkang E Way, Singapore 544886, Singapore; 2 Department of Neurology, National Neuroscience Institute, Singapore General Hospital Campus, Outram Road, Singapore 169608, Singapore; li.wei.shan@singhealth.com.sg

**Keywords:** young-onset dementia, fvAD, bvFTD, primary psychiatric disorder

## Abstract

**Background and Clinical Significance**: In our case study, the patient experienced approximately a year-long delay in her diagnosis, where her initial diagnosis was mistakenly a primary psychiatric disorder, resulting in undue stress on her family. The aim of this case study is to raise awareness of frontal variant Alzheimer’s dementia (fvAD) and to increase knowledge amongst clinicians about this disorder, its management and the need for long-term follow up in specialized clinics. **Case Presentation**: In January 2023, a 56-year-old woman first presented with a 4-month history of worsening cognitive symptoms with considerable overlapping mood symptoms. Her Mini-Mental State Examination (MMSE) score was 20/28, whereas her Frontal Assessment Battery (FAB) score was 6/18. Upon neuropsychological evaluation, she demonstrated multidomain cognitive deficits, where impairments were most prominent in executive dysfunction, learning, memory and semantic fluency. There was evidence of progressive neurodegenerative changes, with brain MRI (April 2024) showing predominant bilateral frontal and parietal volume loss, sparing the occipital and temporal lobes. Amyloid positron emission tomography (PET) was diffusely positive. A diagnosis of fvAD (frontal variant Alzheimer’s dementia) with BPSD was made. Other differential diagnoses included a major neurocognitive disorder due to multiple etiologies (AD and dementia with Lewy bodies (DLB)), frontotemporal dementia (bvFTD), primary progressive aphasia (PPA) and the psychiatric disorder of pseudodementia secondary to a mood disorder. **Conclusions**: This case presented significant challenges given the atypical neuropsychological profile and the complexity of the symptom presentation with significant neuropsychiatric overlay. The preliminary research findings underscore the complexity of fvAD, warranting future research using fundamental approaches.

## 1. Introduction

Frontal variant Alzheimer’s disease is considered a rare form of Alzheimer’s disease which is frequently misdiagnosed. This picture is further complicated by the overlapping presentation of fvAD and behavioral variant frontotemporal dementia (bvFTD). Up to 40% of patients diagnosed with FTD are found to have an AD-like pathology upon post-mortem examination. It is estimated that fvAD accounts for approximately 2–3% of all AD cases [1,2]. However, due to its rarity, most epidemiological studies either group fvAD with other atypical AD variants or misclassify it as FTD. Consequently, the exact epidemiological data for fvAD remain unclear.

In this case, our patient experienced an approximately year-long delay in her diagnosis, as her condition was initially mistaken for a primary psychiatric disorder, resulting in undue stress on the family who could not understand why their loved one was struggling. Misdiagnosis also has significant effects on the caregivers’ burden, on long-term planning (with regard to finances, healthcare decisions) and on care plans. The aim of this case study is to raise awareness of frontal variant Alzheimer’s disease (fvAD), as well as the diagnostic challenges discussed and the need for long-term follow up with specialized clinicians and to increase the knowledge amongst clinicians about this disorder [3].

## 2. Case Presentation

In January 2023, a 56-year-old woman presented with a 4-month history of worsening cognitive symptoms. Her family reported increased distractibility, difficulty using phone applications, repetitive behavior, forgetfulness (misplaced wallet/phone/bus card, failure to recall distant past and recent events) and difficulty initiating and completing tasks. These symptoms were a significant departure from her previous cognitive abilities. Concurrently, there were considerable overlapping mood components, where she was described as being more anxious than usual, which appeared to have begun abruptly following a negative workplace experience in August 2022. There was a change in her job role and she was unable to keep up with the fast-paced job and her new superior was allegedly verbally abusive towards her, berating her in public. She was hugely impacted by this incident and this led to her eventual resignation from work in Jan 2023.

As for her other pertinent personal history, she was married with two children. The patient had completed her education to O-level (equivalent to the 10th grade) and had an employment history as a factory worker and, most recently, a sales assistant. Premorbidly, she was described as timid and introverted, exhibiting some dependent personality traits, such as difficulty making decisions and initiating activities independently and a low self-confidence. A significant family history of dementia, specifically in her father, was noted. No past psychiatric history was reported.

Throughout 2023, the patient experienced intermittent periods of mood disturbances characterized by mixed emotions (anxiety, low mood, anger) which were triggered by difficulties completing tasks, negative thoughts and feelings of helplessness. She exhibited tearfulness during outpatient consultations and was reported by her family to be anxious due to forgetfulness, manifesting as repetitive checking and repacking behaviors. From January to April 2023, she was seen by several different psychiatrists and neurologists at our institution, which complicated her management. Our initial working diagnosis for her was mixed anxiety and depression and cognitive impairment which should be further evaluated. She was started on sertraline for her mood disturbances and referred for an outpatient neuropsychological assessment. Despite optimizing the sertraline dose to 200 mg daily, her mental state did not significantly improve. From April to December 2023, her outpatient care was consolidated under a single psychiatrist. Her cognitive decline continued, with increasing word-finding difficulties and occasional episodes of blank staring. Her husband noticed functional decline, including difficulty with simple tasks such as laundry and a loss of independence regarding cooking, grocery shopping and public transportation. In January 2024, her daughter reported instances of her becoming lost while alone outside of the home and a decreased ability to visit her mother independently.

Ongoing difficulties led to the patient’s first psychiatric admission in April 2024. Upon psychiatric assessment, it was noted that she was frequently dazed and unable to elaborate on her difficulties despite prompting and encouragement. Nursing staff observations through qualitative assessments revealed fluctuations in her mental state, with periods of dazedness in the morning followed by apparent normalcy in the evening. In the preceding month, her family reported odd behaviors (e.g., misplacing clothing, showering with pants on), agitation (temper outbursts), hallucinatory experiences (visual and sensory presence hallucinations) and resistance to care (refusing medication). The hallucinatory experience was characterized by her seeing her mother in the lift lobby when she was not there. There were no aggressive or self-harming/suicidal behaviors. Collateral history was obtained from her family during the inpatient stay. The discrepancy in family reports added to the diagnostic difficulty: her husband described cognitive symptoms as being predominant, whereas her sister reported otherwise (predominant mood symptoms).

Based on the occupational therapist (OT) assessment, she required supervision in some basic activities of daily living (b-ADLs) and assistance for all instrumental activities of daily living (I-ADLs). She required prompting to attend to her toilet and dressing needs, although she was able to carry out the tasks herself. She was no longer able to manage her medication or finances independently.

Her outpatient neuropsychological assessment findings, outlined in Table 1, revealed significant multidomain cognitive deficits, primarily affecting executive function, learning, memory and semantic fluency, with limited effects on visuospatial/visuo-constructional and language domains. However, the findings were effort-dependent and inconsistent.

Her cognitive decline was further supported by other cognitive testing. In early 2023, she scored 20/28 on the Chinese Mini-Mental State Examination (CMMSE), with deficits in orientation, attention, recall and three-step command. The Montreal Cognitive Assessment (MOCA) could not be completed due to her inability to engage. In April 2024, her Frontal Assessment Battery (FAB) score was 6/18. Given her proficiency in Mandarin, the CMMSE, a validated tool with age- and education-adjusted cut-off values, was used for cognitive assessment [4].

**Figure 1:**
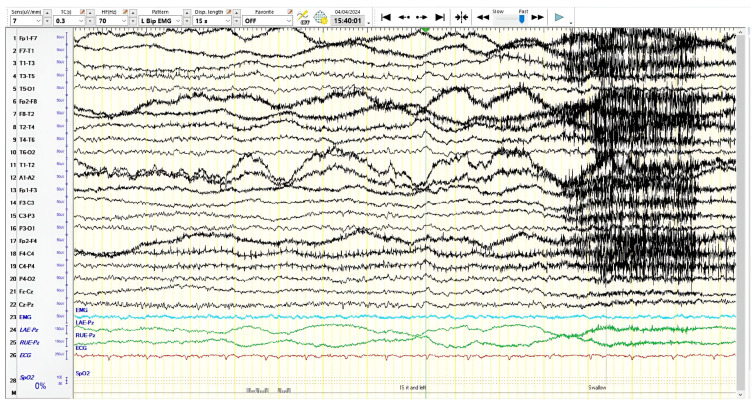
The EEG showed intermittent slow, regional, independent results in the left and right temporal regions, as well as intermittent, generalized, slow, results which suggests bilateral temporal cortical electrophysiological dysfunction and mild diffuse encephalopathy, respectively. The overall EEG features were non-specific in nature.

Neurological examination was normal. Her syphilis/HIV serology, serum folate and vitamin B12, thyroid function, full blood cell count, renal panel and liver function were unremarkable. Electroencephalography was performed in April 2024 and the results were suggestive of mild diffuse encephalopathy and bilateral temporal cortical electrophysiological dysfunction (Figure 1). An occipital sharp transient was observed once during the entire recording, the clinical significance of which is uncertain.

**Figure 2:**
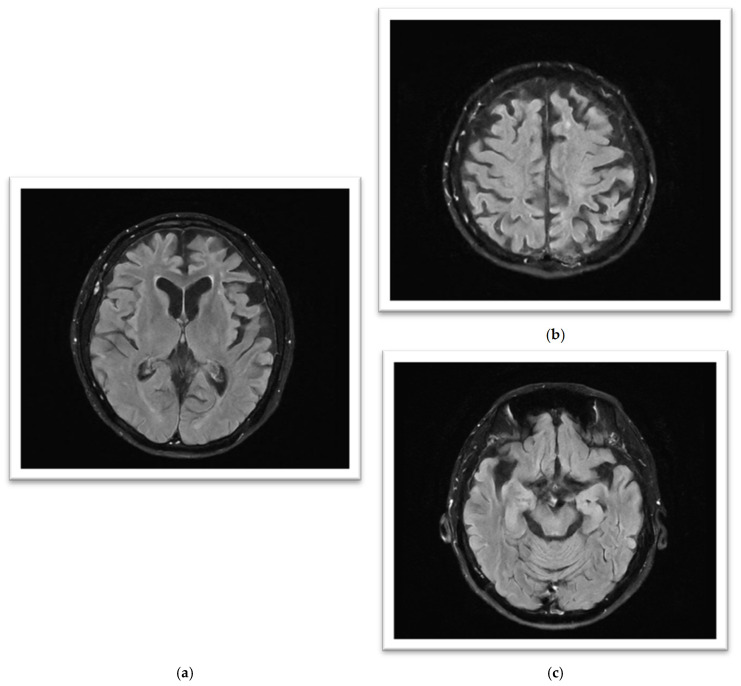
(**a**–**c**): MRI FLAIR sequence (from left to right): (**a**) significant symmetrical gyral thinning in frontal and parietal lobes; (**b**) significant symmetrical gyral thinning in frontal lobes, mostly sparing the occipital lobes; (**c**) reasonably preserved hippocampal volumes bilaterally.

**Figure 3:**
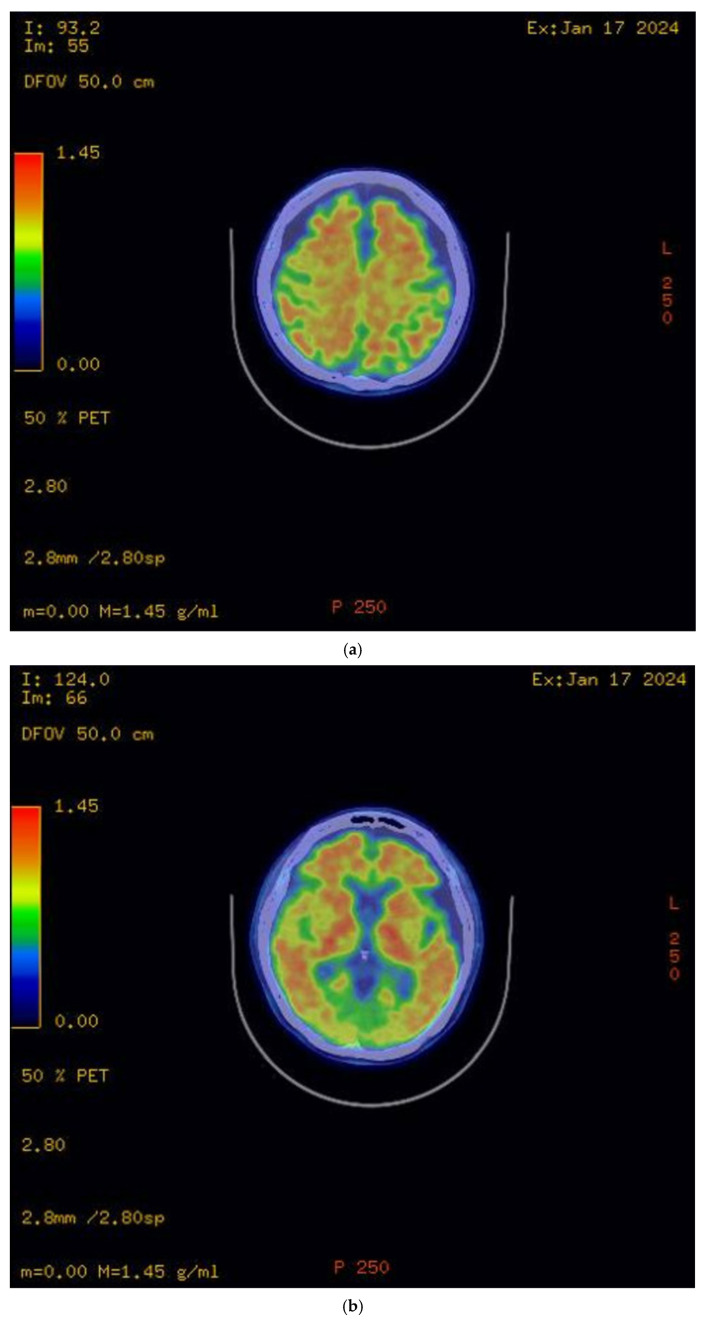
Amyloid PET scan. (**a**) Radiotracer uptake detected in bilateral frontal and parietal lobes; (**b**) radiotracer uptake detected in bilateral temporal lobes.

The initial brain MRI conducted in March 2023 demonstrated global cerebral involution with equivocal frontoparietal predilection and relative medical temporal lobe sparing. Global cortical atrophy (GCA) grade 1–2 was reported, showing mild to moderate brain atrophy with a reduced gyri volume, increased sulci and moderate ventricular dilatation [5]. There was evidence of progressive neurodegenerative changes in the brain MRI findings one year later; the second brain MRI in April 2024 showed predominant bilateral frontal and parietal volume loss, sparing the occipital and temporal lobes. A medial temporal lobe atrophy score (MTA) of grade 1 was reported (Figure 2). Amyloid positron emission tomography (PET) (Figure 3) confirmed the presence of amyloids, a hallmark of Alzheimer’s disease. While a lumbar puncture to measure cerebrospinal fluid (CSF) biomarkers (Aβ1-42 and tau) was offered, the family declined due to the invasive nature of the procedure.

The patient was treated with several psychotropic medications to target her mood and potential apathy. However, she experienced side effects that limited their use. Mirtazapine (7.5 mg nightly) worsened her urinary incontinence and up to 300 mg of bupropion daily was associated with abnormal movements. Olanzapine was started for disorganized and hallucinatory behavior. Nursing staff observations described that she was gesturing to the air, laughing and talking irrelevantly to herself about plucking a flower from a plant. In addition, new symptoms emerged: she was found snacking in the middle of the night, which was very unusual compared to her baseline behavior. She also experienced one episode of abnormal movement one day after the bupropion dose was reduced from 300 mg to 150 mg. The episode was described as her staring up at ceiling and drooling, followed by shaking her head and upper limbs in a rhythmic manner. Repeated brain CT and EEG results were unremarkable. Neurological consultation did not support antiseizure medication, as the episode was considered atypical for a seizure and potentially functional in nature. No further similar episodes occurred.

The neurologist revised the diagnosis to frontal variant Alzheimer’s dementia (fvAD) with behavioral and psychological symptoms of dementia (BPSDs) based on the clinical history, neuropsychological assessments and neuroimaging findings. A multidisciplinary team of neurologists and psychiatrists at the institution concurred with this diagnosis.

The family was informed of the diagnosis, management plan and prognosis through a joint family conference. Non-pharmacological interventions via multidisciplinary support were employed, while the family and caregivers were educated about strategies for behavioral management, including cognitive stimulation, communication strategies to avoid cognitive overloading and the TANGO approach (tender, acknowledge, no interruptions, get into simple conversation, optimize stimuli) to communicate with the patient. In addition, the family was given access to resources from Dementia Singapore for caregiver support and a lasting power of attorney (LPA) was submitted for long-term planning. A social worker was involved in discharge planning and to provide the patient and family with financial assistance through ComCare Short-to-Medium-Term Assistance (SMTA), Social Service Office financial support and care subsidies/funds such as care shield life. The family declined dementia daycare due to costs. The patient was eventually discharged home with a helper and an interim caregiver service (ICS). Her medication upon being discharged included 10 mg Olanzapine nightly, 200 mg sertraline daily, 10 mg Donepezil nightly and 5 mg Memantine twice daily.

**Figure 4:**
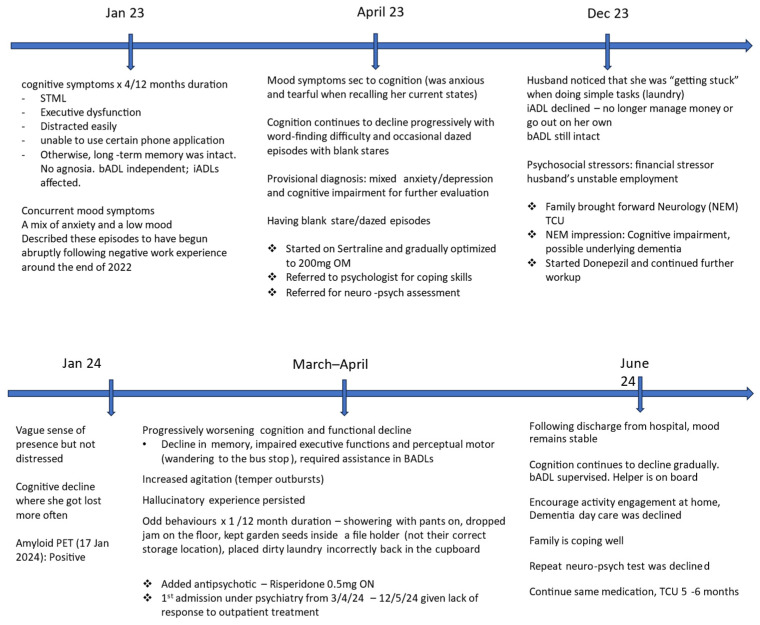
Timeline of presentation.

At the 3–4-month follow-up, her husband reported that her mood had stabilized. However, her cognitive and functional abilities continued to deteriorate and she required supervision for all basic activities of daily living (ADLs). While a repeat neuropsychological assessment was discussed, the family declined due to financial constraints. A follow-up appointment was scheduled six months later to monitor her cognitive progression. Figure 4 provides a timeline summarizing the case.

## 3. Discussion

This case presented significant challenges given the atypical neuropsychological profile and complexity of the symptom presentation, with significant neuropsychiatric overlay. Differential diagnoses included a major neurocognitive disorder due to multiple etiologies (Alzheimer’s disease and dementia with Lewy bodies (DLB)) given the potential apathy, fluctuating attention/cognition, possible visual hallucinations and cognitive impairments in learning and memory. Frontotemporal dementia (bvFTD) was another consideration due to the apathy and impaired semantic fluency. However, the absence of significant behavioral disinhibition, socially inappropriate behaviors and other speech abnormalities rendered a behavioral variant of FTD or primary progressive aphasia (PPA) less likely. Recurrent vivid visual hallucinations, parkinsonism and rapid eye movement (REM) sleep behavior disorder, which are characteristic of DLB, were also absent.

The patient’s diagnosis was revised from a primary psychiatric disorder to frontal variant Alzheimer’s disease (fvAD) based on her clinical history, neuropsychological assessment and neuroimaging findings. The positive family history of dementia and significant cognitive decline in the absence of a long psychiatric history supported this diagnosis. Additionally, her mood symptoms were not pervasive, as evidenced by the variability in the neuropsychological assessment findings. She did not exhibit psychopathology consistent with psychotic depression. The neuropsychological assessment showed significant impairment in multiple cognitive domains, including a severely impaired executive function sparing the visuospatial/vasoconstriction and language domains. The overall findings of her neuropsychological assessment were different from those of typical AD patients, which usually predominantly show memory impairment.

The diagnosis of Alzheimer’s disease (AD) in this case was based on the revised criteria for the diagnosis and staging of Alzheimer’s disease: Alzheimer’s Association Workgroup [6]. AD is defined by its biology, and therefore, a biomarker that can accurately detect AD neuropathologic changes (ADNPCs) or a validated surrogate is sufficient to establish the diagnosis of this disease. Diagnosing AD via abnormal amyloid PET (or biofluid Core 1 biomarkers validated against amyloid PET) in symptomatic individuals in this case is sufficient and meets the criteria based on the recommendations by the Alzheimer’s Association Workgroup. Amyloid PET scans and CSF AD biomarkers are core biomarkers commonly used for AD diagnosis. While both are valuable, amyloid PET scans are less invasive than CSF biomarker testing, making them a more acceptable option for some patients. Blood-based biomarker testing is another less invasive alternative, and advancements in technology have led to some blood-based biomarkers achieving a sensitivity and specificity comparable to CSF-based biomarkers. However, due to the limited availability of blood-based biomarker testing at our facility, these diagnostics are currently inaccessible onsite. Furthermore, sending samples to overseas laboratories would result in significant delays and substantial costs—a significant concern for the patient and her family.

Given that AD is the most common cause of cognitive impairment globally, even among younger individuals, it was a reasonable consideration, especially in the context of a predominantly neuropsychiatric presentation. Atypical variants of AD, such as the frontal variant, can often present with neuropsychiatric symptoms such as irritability, apathy, anxiety, depression and anosognosia. These symptoms can sometimes lead to an initial misdiagnosis of a primary psychiatric disorder rather than a neurodegenerative condition.

As shown in the previous literature, there may be significant overlap in psychiatric, neurological, cognitive and even neuroimaging changes in young-onset dementia syndromes and primary psychiatric disorders, causing diagnostic uncertainty and delay. Based on a study by Tsoukra et al. [7], 49 of 127 patients (39%) had their initial diagnoses revised during the follow-up period. This included changing from a diagnosis of a neurodegenerative disease to another neurodegenerative or neurological condition (24%), from a neurodegenerative diagnosis to a psychiatric diagnosis (10%), or from a psychiatric diagnosis to a neurodegenerative diagnosis (5%). Alzheimer’s disease-type dementia was the most stable diagnosis over time [7].

Frontal variant Alzheimer’s disease (fvAD) can manifest in either behavioral or dysexecutive syndromes [8]. While some studies suggest these are distinct entities, others comparing fvAD and bvFTD have yielded conflicting results [9]. Symptoms common to both conditions are executive dysfunction and apathy, which were observed in our patient. Atrophy is typically more prominent in the frontal and anterior temporal lobes, often asymmetrically, but parietal involvement is less typical in neuroimaging findings. There is a prevailing difficulty in differentiating between fvAD and bvFTD. A case series of six patients showed that the clinical criteria for bvFTD can correspond to histologically confirmed AD, whereas another autopsy series of 60 patients with a clinical diagnosis of bvFTD showed that 7% had histologically confirmed AD and 10% had a combination of both diseases. Recent studies that have compared the clinical symptoms of fvAD and bvFTD are conflicting. BvFTD may lead to memory issues in the earliest stages of the disease, making the distinction between the two pathologies tricky. Early executive disorders have long been specifically attributed to patients with bvFTD; however, studies show that early executive function deficits may exist in AD [10]. Currently, there are no consensus criteria for dysexecutive or behavioral variant AD, although preliminary criteria have been proposed for clinical practice [11]. 

## 4. Conclusions

Differentiating between young-onset dementia and primary psychiatric disorders can be challenging, often leading to changes in diagnosis over time. Patients whose initial diagnosis was neurodegenerative but later changed to psychiatric are more likely to have a history of mental health issues. Conversely, those with an initial psychiatric diagnosis that shifted to neurodegenerative typically experience early-onset psychiatric symptoms. It is important to note that late-onset psychiatric symptoms may be a harbinger of a neurodegenerative disease.

The significant diagnostic challenges highlighted in this case emphasize the importance of a comprehensive evaluation process for young-onset dementia, including gathering a detailed history, neuropsychological assessments, neuroimaging studies and, when possible, biomarker testing. Early and accurate diagnosis is crucial for timely interventions, the management of neuropsychiatric symptoms and patient and family education and support. Collaboration between neurologists, psychiatrists and other healthcare professionals is essential to ensure optimal patient care.

Differentiating fvAD and other neuropsychiatric disorders remains a complex task that requires continued extensive research conducted on typical cohorts in high-quality studies. We envision a comprehensive system incorporating imaging, biological markers—particularly blood-based biomarkers—and neuropsychological and clinical profiles, taking both a dimensional and a longitudinal approach, along with practical application of the system in the real world. With the increasing capabilities of artificial intelligence (AI) on the horizon, a streamlined and accelerated process integrating clinical, neuropsychological, radiological and biochemical data could offer a highly accurate and cost-effective approach for detection of early-stage or even subclinical fvAD in individuals.

## Figures and Tables

**Table 1:** Neuropsychological assessment and results.

Cognitive Domain	Neuropsychological Test	Brief Description of the Outcome
Perceptual motor	Repeatable Battery for the Assessment of Neuropsychological Status—Singapore version (RBANS-SG) Oral Trail-Making Test Part A	Normal visuospatial/visuoconstructional functioning
Language	Modified 30-item Boston Naming Test Common Objects Memory Test Controlled Oral Word Association Test Animal Fluency Cookie Jar Picture Description Test	Variable While nominal function/repetition were intact, semantic fluency was severely impaired Description of a pictorial scene was adequate
Memory and learning	Story Memory and Recall List Learning and Recall/Recognition Figure Copy and Recall, Coding Sentence Repetition Test Oral Trail-Making Test Part A	Severely impaired, with poor scores across tasks of verbal and visual memory, including those of learning, recall and recognition
Executive function	Victoria Stroop Test and Wechsler Adult Intelligence Scale, 4th Edition (WAIS-IV) Trail-Making Test Frontal Assessment Battery (FAB)	Severely impaired The FAB test was discontinued as she was unable to follow some instructions
Complex attention	Digit Span (select subtests) Processing speed tests	Inconsistent and variable immediate verbal attention and working memory Unable to count backwards from 10, getting stuck at 7; prompts were of no help Processing speed tests were discontinued due to her difficulties in understanding the task requirements or being able to perform the necessary functions for task completion
In summary, on neuropsychological evaluation, her presentation upon interview and testing, as well as her performances in effort tests, was indicative of non-credible cognitive symptom presentation and fluctuating levels of motivation.		

## Data Availability

Data availability requests may be sent to the first and corresponding author if needed due to privacy concerns.
